# National Hospitalization Trends Among Centenarians in the United States

**DOI:** 10.1093/geroni/igaa057.1207

**Published:** 2020-12-16

**Authors:** Sylvia Twersky, Adam Davey

**Affiliations:** 1 The College of New Jersey, Ewing, New Jersey, United States; 2 University of Delaware, Newark, Delaware, United States

## Abstract

Continued increases in life expectancy mean than an unprecedented number of individuals are reaching centenarian status, often with complex health concerns. Little research has addressed acute care experiences of centenarians. To address this knowledge gap, we analyzed 10 years of hospital admissions data from a total of 52618 centenarians (aged 100 to 115 years) in the nationally representative National Inpatient Study (NIS) with the aim of characterizing centenarian (mean age 101.4 years, 80% women, 24.2% non-White, 91.3% Medicare, 72.2% emergencies, 18.6% urgent) hospitalizations. Mean length of stay (LOS) decreased from 6.1 to 5.1 days (2000-2009). Over the same period, mean total charges rose from $13373 to $25026; they were $4478 lower for women than men and $4032 more for centenarians age 105 years and older than ages 100 to 104, despite comparable LOSs. Mean LOS was approximately 0.7 days longer for non-Whites than Whites, and mean total charges were approximately $6531 higher for non-Whites than Whites. The 10 most common admission diagnoses were consistent with frailty and end stage cognitive impairment including pneumonia, congestive heart failure, urinary tract infections, fracture of neck of femur, septicemia, fluid and electrolyte disorders, aspiration pneumonitis, acute cerebrovascular disease, acute myocardial infarction, and gastrointestinal hemorrhage, and 10.0% of centenarians died in hospital. We link individual-level data with hospital and state characteristics over the same time period in order to provide a more detailed analysis of individual and system-level characteristics that contribute toward health disparities in hospitalizations and health outcomes among centenarians.

